# Short‐Segment Fixation of Thoracolumbar Fractures with Incorporated Screws at the Level of Fracture

**DOI:** 10.1111/os.12590

**Published:** 2020-01-08

**Authors:** Hassan Fathy El Behairy, Ashraf M Abdelaziz, Ayman K Saleh, Faisal Ahmed Hashem Elsherief, Ibrahim Elsayed Abdellatif Abuomira, Ahmed Ibrahim Elkawary, Wael Aldahshan, Wael Sh Mahmoud

**Affiliations:** ^1^ Alzhraa University Hospital, Faculty of Medicine for Girls Al‐Azhar University Cairo Egypt; ^2^ Surgery Department, College of Medicine Prince Sattam Bin Abdulaziz University, Al‐kharj, al Riyadh Saudi Arabia; ^3^ Al‐Azhar University Cairo Egypt

**Keywords:** Intermediate screw, Short‐segment fixation, Thoracolumbar fracture

## Abstract

**Objective:**

To evaluate the effect of including the fractured vertebra in the short‐segment fixation of thoracolumbar (TL) fractures.

**Methods:**

A total of 32 patients with thoraco‐lumbar fractures, selected between August 2013 and February 2016, were managed by short‐segment fixation with screws at the level of the fracture, and decompression was performed only for patients with neurological deficits. The patients' functional outcome was assessed using the visual analogue scale (VAS) score for pain and the American Spinal Injury Association (ASIA) score for neurological condition. All patients were followed up with radiographs.

**Results:**

Patients with complete neurologic deficits (*n* = 3) did not show any neurologic recovery. All ASIA B patients improved to ASIA C. Five ASIA C patients improved to ASIA E. The remaining five ASIA C patients improved to ASIA D. All ASIA D patients improved to ASIA B. At the final follow‐up examination, the mean anterior vertebral height was 21 ± 5 mm, indicating no significant height loss during the follow‐up period.

**Conclusion:**

Short‐segment fixation of TL fractures with inclusion of the fracture level into the construct offers good correction of segmental kyphosis, vertebral wedging, and vertebral height loss.

## Introduction

The thoracolumbar (TL) region of the spine ranges, by definition, from T_11_ to L_2_ inclusively, and nearly 60%–70% of all traumatic spinal fractures occur in the TL region. Biomechanical studies have demonstrated that in the upright position, 80%–90% of the axial compression force is absorbed by the anterior part of the spine body and intervertebral disc, whereas the posterior articular facets absorb the remaining 10%–20%^1^.

Several classification systems of TL spine fractures designed to provide standardization for spinal surgery have been defined in the literature. These systems include: the Boehler classification, 1930; the Watson–Jones classification, 1938; the Nicoll classification, 1949; the Holdsworth classification, 1963; the Kelly and Whitesides classification, 1968; the Denis classification, 1983; the McAfee classification, 1983; the Ferguson‐Allen classification, 1984; the McCormack classification (load‐sharing classification), 1994; and the AO classification, 1994^1^.

The most recently established classification system is the Vaccaro classification (Spine Trauma Study Group Thoracolumbar Injury Classification and Scoring System [TLICS]), which was developed in 2005 with the goal of improving the shortcomings of previous systems, facilitating communication among researchers and clinicians, and optimizing patient care (Table 1). This classification takes into account the fracture mechanism, the completeness of the posterior ligamentous complex, and the neurological status of the patient. According to this classification system, in cases with fewer than three points, surgical intervention is not suggested; in cases with four points, operative or non‐operative intervention is suggested; and in cases with five points or more, operative intervention is suggested^2^.

**Table 1:** Vaccaro classification (Spine Trauma Study Group Thoracolumbar Injury Classification and Scoring System [TLICS])

	Points
Fracture mechanism	
Compression fracture	1
Burst fracture	1
Rotational fracture	3
Splitting	4
Neurological involvement	
None	0
Nerve root	2
Medulla spinalis, conus medullaris, incomplete	3
Medulla spinalis, conus medullaris, complete	2
Cauda equine	3
Posterior ligamentous complex	
Intact	0
Possibly injured	2
Injured	3

Axial load injuries, such as compression and burst fractures, involving the TL junction without associated neurologic injury, are often managed nonoperatively with bracing and early mobilization. Operative intervention may be warranted in certain fractures, especially in those with associated neurologic injury and/or perceived mechanical instability. Patient factors, such as multi‐trauma and inability to tolerate bracing, may also lead to surgical treatment in cases that would otherwise be non‐operative. In these situations, operative intervention allows for neural element decompression when needed, restoration of sagittal alignment, and early patient mobilization. The appropriate surgical technique for unstable TL compression and burst fractures remains controversial^3^.

Short‐segment fixation of the fracture level has replaced the traditional long‐segment instrumentation to decrease the number of motion segments sacrificed during the fusion process. However, when significant disruption of the load‐sharing anterior column occurs, the simple one level above and one level below short‐segment fixation does not ensure adequate stability and results in a poor reduction in kyphotic deformity and the occurrence of instrument failure. Such outcomes necessitate more extensive approaches, such as anterior reconstruction *via* an anterior approach or a posterior method.

Several studies have shown that by inserting screws at the fracture level, the construct becomes biomechanically stronger^4^.

A balance between avoiding anterior surgeries and preventing failure in posterior surgeries can be achieved by increasing the number of fixation points along the posterior construct using intracorporeal filling techniques and “intermediate” screws at the level of the fracture. The use of intermediate screws is a novel technique for increasing the fixation strength of a posterior short‐segment construct^5^.

Biomechanical studies have shown that the addition of a screw at the level of the fracture in a short‐segment fixation (posterior fixation including fractured vertebra [PFFV]) increases the stiffness of the construct and protects the anterior column during loading^6^.

The purpose of this prospective study was: (i) evaluation of the efficacy of screw placement at the fracture level for the correction of deformity; (ii) maintenance of correction; and (iii) prevention of failure of the fixation in TL unstable fractures.

## Materials and Methods

Between 2013 and 2016, 32 patients were admitted to the Orthopedic Department at Al Zahraa University Hospital. The patients presented with unstable TL fractures between T_10_ and L_3_ and were treated with short‐segment fixation (PFFV).

### *Inclusion and Exclusion Criteria*

The inclusion criteria were as follows: (i) patients aged from 20 to 50 years; (ii) patients with unstable TL fractures between T_10_ and L_3_; (iii) kyphosis progressing to 20% or more; (iv) more than 50% loss of vertebral body height; (v) more than 50% canal involvement; and (vi) patients treated with short‐segment fixation.

Exclusion criteria: (i) osteoporotic patients; (ii) patients with fracture‐dislocation; and (iii) patients with broken pedicles or unstable pedicles.

### *Radiographic Study*

All patients underwent preoperative plain X‐rays, computed tomography (CT), and magnetic resonance imaging (MRI) of the TL spine to evaluate the fracture. A fracture of any of the pedicles was not considered a contraindication to screw placement. The load sharing score was calculated based on the scoring system described by McCormack *et al*.^7^. The neurological status of each patient was recorded based on the American Spinal Injury Association (ASIA) classification criteria (Table 2).

**Table 2:** American Spinal Injury Association (ASIA) classification of spinal cord injury^8^

**Grade A:** Complete lack of motor and sensory functions below the level of injury (including anal area).
**Grade B:** Some sensation below the level of injury (including anal area).
**Grade C:** Some muscle movement spared below the level of injury but 50% of muscle below the level of injury cannot move against gravity.
**Grade D:** Most (more than 50%) of the muscles that are spared below the level of injury are strong enough to move against gravity.
**Grade E:** Normal function.

### *Surgical Technique*

**Figure 1:**
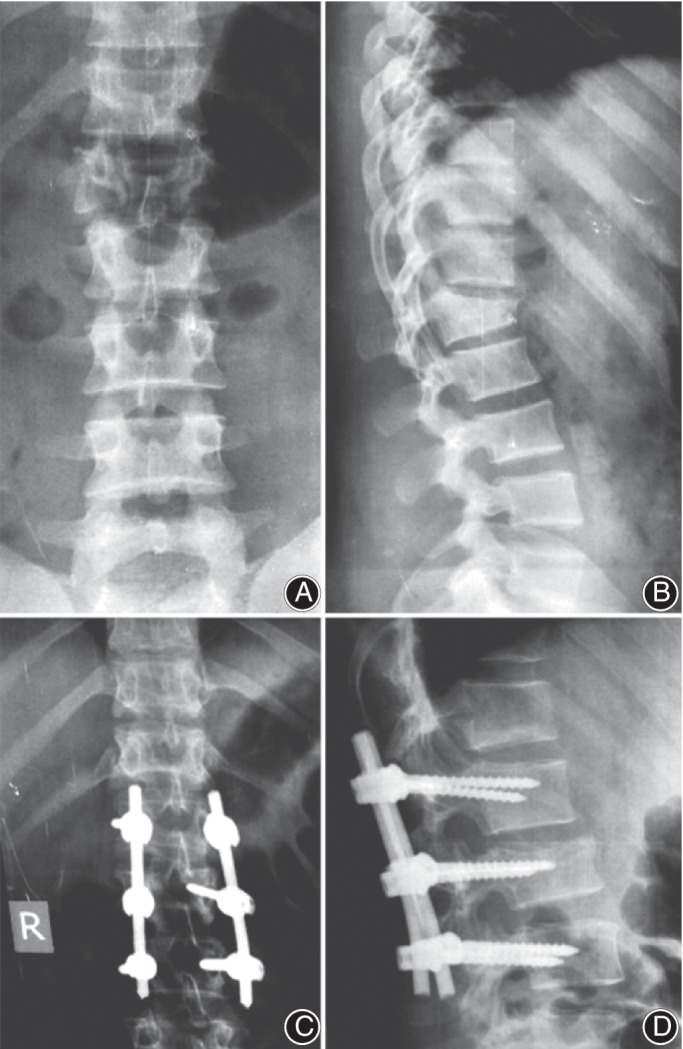
Preoperative (A) anteroposterior (AP) and (B) lateral views, showing type 2 burst fracture L_2_. Postoperative (C) AP and (D) views, showing fracture L_2_ fixed by pedicular screw from L_1_ to L_3_ with screws at fractured L_2_ with the incorporation of an intermediate screw.

**Figure 2:**
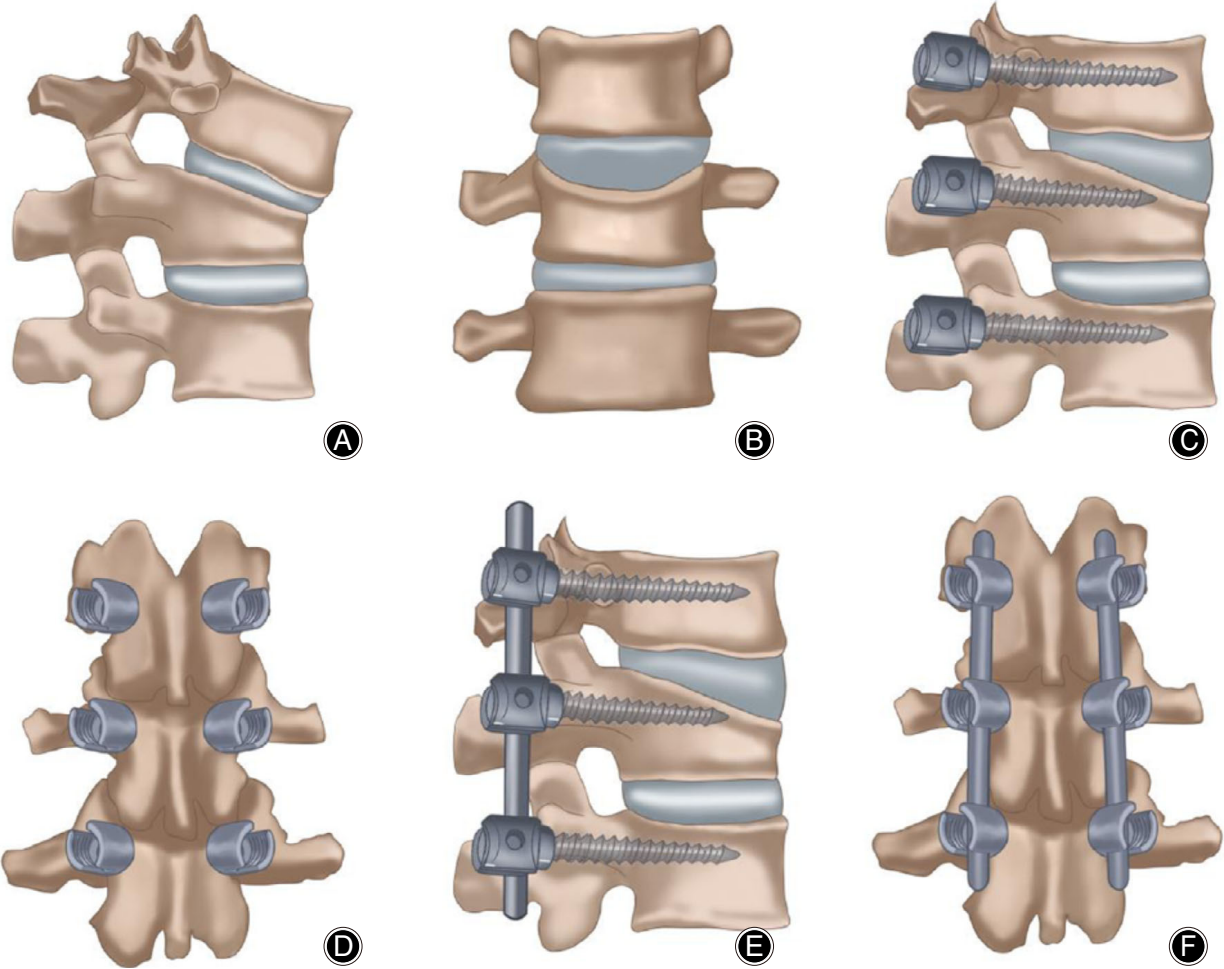
(A) Lateral and (B) anteroposterior (AP) views showing burst fracture; (C) lateral and (D) AP views showing burst fracture fixed by pedicular screws at fractured level and level above and level below; (E) lateral and (F) AP views showing burst fracture fixed by pedicular screws at fractured level and level above and level below and rod fixation.

A standard posterior midline approach was performed, and pedicle screws were inserted into the vertebra cephalad and placed caudal to the fracture. The screw size was chosen according to the size of vertebra (5.5–6.5 mm in diameter × 40–45 mm in length; rods were typically 5–6 mm), and the screws were inserted at a level above and a level below the injury and into the pedicles of the fractured vertebrae (intermediate screws) using a free‐hand technique. The intermediate screw heads were left slightly proud to act as a push point and achieve a reduction of kyphosis. Intermediate screws were inserted in both pedicles of the fractured vertebra (Figs. 1 and 2); however, if the pedicle walls were broken, no screws were inserted into that pedicle. We achieved complete reduction of kyphosis or restored vertebral height by rod overcontouring or distraction. In fractures with neurologic deficits or spinal canal compromise greater than 50%, a decompressive laminectomy was performed. Anterior decompression surgery was not performed in any of the patients.

The degree of kyphosis correction and the position of the screws were assessed by postoperative radiographs. In patients with coexistent fractures at adjacent levels, additional vertebrae were fixed. All patients were periodically followed up with clinical and radiological evaluations. Radiographs were performed at 3, 6, 12, and 24 months after surgery.

### *After Surgery*

Stitches were removed approximately 10–14 days after surgery. The patients were allowed to move freely in a TL rigid brace the day after surgery. Sitting was permitted 2 weeks after surgery. All patients were advised to actively move their legs to strengthen their abdominal muscles and thighs.

### *Outcome Measures*

The patients' demographic, clinical, and injury details (age, sex, mode of injury, time to surgery from injury occurrence, associated injuries, preoperative and postoperative neurologic status, and preoperative complications) were studied and the limitation of motion was estimated during follow‐up examination. The patients' functional outcome was assessed using the visual analogue scale (VAS) score for pain.

#### *American Spinal Injury Association Score*

The American Spinal Injury Association Score (ASIA) is commonly used to quantify neurological deficits. It is based on motor and sensory scores, neurological levels, a completeness criterion, zones of partial preservation, and an impairment scale. The impairment scale is as follows: (i) no sensory or motor function is preserved, (ii) incomplete sensory but no motor function; (iii) incomplete motor function is preserved below the neurological level and more than half of the key muscles below the neurological level have a muscle grade less than 3; (iv) incomplete motor function is preserved below the neurological level, and at least half of the key muscles below the neurological level have a muscle grade greater than or equal to 3; and (v) sensory and motor function are normal.

#### *Radiographic Measures*

In postoperative radiographs, the following parameters were studied to evaluate the efficacy of fracture‐level screw incorporation in unstable TL fractures and to examine the criteria for implant failure: segmental kyphosis angle, wedge angle, anterior vertebral height loss, posterior vertebral height loss, screw breakage, screw pullout, pen‐implant loosening, and rod breakage. The adjacent vertebra above and below the fixation were also assessed (Table 3).

**Table 3:** Demographic distribution and presentation of cases

No.	Age (years)	Sex	Time interval between injury and admission (d)	Time interval between injury and surgery (d)	Injury level	ASIA
1	38	M	0	0	L_1_	C
2	51	M	1	1	T_12_	F
3	24	M	0	1	L_2_	E
4	45	M	1	1	T_12_	B
5	23	F	0	0	L_1_	F
6	60	M	4	10	T_11_	F
7	18	M	0	0	T_12_	C
8	57	M	2	7	L_1_	F
9	23	F	0	2	L_2_	E
10	45	M	1	1	T_12_,L_1_	A
11	20	M	0	1	L_1_	B
12	36	M	1	1	L_2_	D
13	49	M	0	1	T_12_	C
14	19	F	2	5	T_11_	A
15	41	M	0	0	L_1_	B
16	22	M	I	2	L_2_	C
17	53	F	0	0	T_12_	C
18	37	M	1	2	L_1_	D
19	43	M	0	1	L_2_	C
20	18	M	4	6	T_12_	E
21	21	F	0	0	T_11_,_12_	C
22	30	M	8	10	L_1_	F
23	50	M	1	2	L_1_	D
24	22	F	0	1	T_12_	A
25	40	M	2	3	L_1_	E
26	44	M	0	1	L_2_	D
27	26	M	1	1	T_12_	E
28	58	F	1	1	L_3_	C
29	33	M	0	1	T_10_,T_11_	D
30	52	F	2	4	T_12_	C
31	31	M	0	0	L_1_	B
32	47	M	1	1	L_1_	C

ASIA, American Spinal Injury Association; F, female; M, male.

### *Statistical Analysis*

Statistical analysis was performed for correlations between the preoperative and postoperative follow‐up results. All data were analyzed using the UPSS 13.0 software for windows (SPSS, Chicago, IL, US).

The patients' functional outcome was assessed using the VAS score for pain and the ASIA score for neurological condition. The significance of the changes in the radiographic parameters between the preoperative and postoperative radiographs (measuring kyphotic angle) was statistically analyzed using Student's *t*‐test.

## Results

### *Patients' Information*

A total of 32 patients with TL fractures were admitted, 17 of whom had other organ injuries, such as long bone fractures or abdominal organ injuries. The mean follow‐up time was 21 months (range, 18–24 months). The preoperative clinical data are presented in Table 2. The mean age of the patients was 36.75 years (range, 18–60 years). The male to female ratio was 3:1. The mean time to operate from the time of injury was 2.09 days. Approximately 84.3% of patients underwent surgery within 48 hours after the injury and all patients underwent surgery within 1 week.

### *Operative Details*

Posterior instrumented stabilization without laminectomy was performed in 10 patients (all ASIA B), and midline decompressive laminectomy with stabilization was performed in 22 patients with neurologic deficits. In 29 patients, only a three‐level fixation was used (one level above, below and at the fracture level). In 3 patients, four levels were fixed because of coexistent fractures at adjacent levels. In 30 patients, the screws were inserted at both pedicles at the fractured vertebra, whereas in 2 patients, the screws were inserted unilaterally because of broken pedicles on the opposite side. The mean operating time was 118 ± 36 min; the mean blood loss was 270 ± 95 mL; and the mean duration of hospitalization was 7 ± 4 days.

### *American Spinal Injury Association Score*

#### *Preoperative Assessment*

Among the vertebral fractures, L_1_ was the most commonly affected vertebra (*n* = 12), followed by T_12_ (*n* = 11), L_2_ (*n* = 6), T_1_ (*n* = 4), L_3_ (*n* = 1), and T_10_ (*n* = 1); 10 patients had normal neurology (ASIA E), 19 had incomplete deficits (ASIA B, C, and D), and 3 had complete deficits (ASIA‐A). Regarding the mechanism of injury, 19 patients sustained falls from height and the remaining 13 patients were injured in road traffic accidents. Twenty‐five per cent of fractures had a load‐sharing classification (LSC) <7, and 75% had a LSC = 7.

#### *Postoperative Results*

Patients with complete neurologic deficits (*n* = 3) did not show any neurologic recovery. Four ASIA B patients improved to ASIA C. Five ASIA C patients improved to ASIA E. The remaining 5 ASIA C patients improved to ASIA D. Five ASIA D patients improved to ASIA B. One patient (3.1%) had a postoperative superficial wound infection and responded to antibiotics. One patient (3.1%) had postoperative implant failure caused by an increased kyphotic angle observed at the 18‐month follow‐up examination; this patient had LCS 8 and ASIA C.

### *Visual Analogue Scale Score*

At the final follow‐up, the average VAS score for back pain was 5 ± 2 (range, 1–4).

### *Radiographic Outcome*

The mean preoperative kyphotic angle was 25° ± 8°, which improved significantly to 9° ± 7° in the immediate postoperative period. A mean loss of 2.5° was observed at the final follow‐up examination. The mean preoperative wedge angle was 23° ± 8°, which was corrected to 10° ± 6° (significant) during the immediate postoperative period. A loss of kyphosis (mean 1.2°) occurred during the follow‐up period, which resulted in a mean wedge angle of 11° ± 6°. The mean anterior vertebral height was 14 ± 4 mm, whereas the value was 27 ± 4 mm in the immediate adjacent normal vertebra. This parameter improved to 22 ± 5 mm during the immediate postoperative period. At the final follow‐up examination, the mean anterior vertebral height was 21 ± 5 mm, indicating no significant height loss during the follow‐up period. The mean posterior vertebral height was 25.5 ± 4.5 mm, whereas this value was 29.5 ± 3.5 mm in the normal adjacent vertebra. After surgery, the mean height improved to 28 ± 3 mm (significant). This height restoration was maintained until the final follow‐up examination (28 ± 2.5 mm).

## Discussion

Short‐segment pedicle screw fixation involving one vertebra above and one vertebra below the fracture level has become the most common method for treating unstable TL fractures^9^.

This approach provides the benefit of decreased involvement of motion segments compared with fixation using longer instrumentation. The advantages include the familiarity and simplicity of the approach and reduced complications compared with anterior surgeries. However, this technique has been criticized because of the risks of implant failure and the progression of symptomatic kyphosis. These outcomes have been mainly attributed to a defective anterior weight‐bearing column of the spine^9^.

In a similar study conducted by Guven *et al*., the authors studied the inclusion of the fracture level in both short‐segment and long‐segment fixation. The authors observed that fracture‐level fixation lowered the rates of correction failure, and this reduction was most significant in short‐segment constructs. The authors concluded that fracture‐level screw combinations can achieve and maintain the kyphosis correction^10^. In addition, in a cadaveric biomechanical study, Mahar *et al*. showed that the insertion of the screws at the fracture level improved the biomechanical stability by providing additional fixation, and segmental fixation with additional screws at the level of the fracture increased the construct stiffness and shielded the fractured vertebral body from anterior loads^11^.

The use of intermediate screws provides the effect of a three‐point fixation of the fractured segment and a better pullout strength because two additional fixation points are provided. This procedure also provides for anatomic continuity that is usually preserved between the pedicles and the articular process or pars interarticularis. Therefore, the screws holding the pedicles at the fractured level are not “floating” and can transmit loads to the adjacent vertebrae through the posterior elements^12^.

Before the development of the LSC, the most widely used fracture classification in the United States was the three‐column classification system described by McAfee *et al*.^13^. After routine CT scanning of fresh spinal fractures was introduced, the identification of middle‐column injuries was simplified, and many American surgeons rapidly followed the suggestion of McAfee *et al*. to use the presence of middle‐column injuries as the sole indication for surgical treatment.

The LSC was developed by McCormack *et al*.^7^ to identify fractures that require supplemental anterior reconstruction. The authors retrospectively analyzed 28 patients who had undergone surgery for TL injuries with Steffee Plates. After a mean follow‐up period of 3 years, the authors observed implant failures in 10 patients, and they identified three important factors in predicting posterior fixation failure and scored each factor on a point system from 1 to 3. The authors observed that anterior vertebral reconstruction is essential in patients with an LSC score of 7 or greater to prevent implant failure^14^.

Wang *et al*. created a bovine LI burst fracture model by axial compressive impacts with differential energy and showed a significant positive correlation between the LSC score and spinal instability^15^.

In our study, 75% of patients had LSC values of 7 and underwent short‐segment fixation using incorporated screws at the level of fracture with good results (1 patient [3.1%] experienced implant failure).

Guven *et al*. observed that short‐segment fixation with a fracture‐level screw combination provided better intraoperative correction and maintenance than short‐segment fixation without fracture‐level screws^10^. In addition, an expectation of high rigidity of the construct, the conservation of additional motion segments caudally for balancing, and the maintenance of active spinal movement have been reported for this approach^16^.

### *Conclusion*

Short‐segment pedicle screw fixation that includes the fractured vertebra achieved a reduction in unstable TL injuries and allowed for good correction of segmental kyphosis, vertebral wedging, and vertebral height loss. In addition, using this approach reduced the number of motion segments sacrificed in the fusion by long‐segment fixation. The radiologic correction achieved is maintained even at the end of 2 years, which is reflected in good functional outcomes without additional complications. Thus, we recommend the insertion of screws into pedicles of the fractured TL vertebra when considering a short‐segment posterior fixation.
